# Verbal Synchrony and Action Dynamics in Large Groups

**DOI:** 10.3389/fpsyg.2016.02034

**Published:** 2016-12-26

**Authors:** Jorina von Zimmermann, Daniel C. Richardson

**Affiliations:** Department of Experimental Psychology, University College LondonLondon, England

**Keywords:** synchrony, behavioral coordination, affiliation, joint action, cooperation

## Abstract

While synchronized movement has been shown to increase liking and feelings of togetherness between people, we investigated whether collective speaking in time would change the way that larger groups played a video game together. Anthropologists have speculated that the function of interpersonal coordination in dance, chants, and singing is not just to produce warm, affiliative feelings, but also to improve group action. The group that chants and dances together hunts well together. Direct evidence for this is sparse, as research so far has mainly studied pairs, the effects of coordinated physical movement, and measured cooperation and affiliative decisions. In our experiment, large groups of people were given response handsets to play a computer game together, in which only joint coordinative efforts lead to success. Before playing, the synchrony of their verbal behavior was manipulated. After the game, we measured group members’ affiliation toward their group, their performance on a memory task, and the way in which they played the group action task. We found that verbal synchrony in large groups produced affiliation, enhanced memory performance, and increased group members’ coordinative efforts. Our evidence suggests that the effects of synchrony are stable across modalities, can be generalized to larger groups and have consequences for action coordination.

## Introduction

Thirty strong legs are rhythmically thrilling the ground. Chests, thighs, and arms become drums and strong voices are forcefully chanting together. Eyes are rolled and tongues are poked out. Before every match, the New Zealand rugby team performs the haka, a traditional Maori war dance composed of rigorous, synchronized movements and fierce, rhythmical chants. Amongst other things, the haka was performed before a battle to demonstrate strength and power and to intimidate the opponent. However, anthropologists and historians have argued for a long time that ‘keeping together in time’ (McNeill, 1995) induces emotional bonding among human groups with significant consequences for interaction and cooperation (von Zimmermann and Richardson, 2015). The haka might scare the enemy on the battlefield or rugby pitch, but it might also strengthen intragroup bonds and have a significant impact on the group’s performance.

Rhythmic and coordinated actions such as marching, dancing, singing, or playing music together have been part of human rituals across all cultures in the world (McNeill, 1995; Codrons et al., 2014), but synchrony is not only a human phenomenon. It can be found everywhere in the natural world as well. For example, cardiac cells fire in synchrony and fireflies flash in unison (Strogatz, 2003; Cabeza et al., 2010). Metronomes automatically synchronize if they are put on a freely moving base (Pantaleone, 2002), and neurons synchronize their activity to allow for coherent percepts and actions (Singer, 1993). Human beings coordinate their postural sway during conversation (Shockley et al., 2009), and their movements during a pendulum swinging task, or while rocking in a chair when visually coupled (Richardson et al., 2005; Richardson M. J.et al., 2007). There seems to be a compelling drive for systems to self-organize in synchrony (Strogatz, 2003), and it has been suggested that human beings possess a fundamental drive to coordinate their actions with the actions of others, as this forms the basis for social connectedness (Marsh et al., 2009).

Social scientists have started to collect empirical evidence for the effects of synchronized human activity and a growing body of research supports the idea that coordinated action can function as ‘social glue’ that binds people together and enhances their willingness to cooperate (Valdesolo et al., 2010). For example, observing synchronous movement increases perceived rapport and interpersonal connectedness between people (Miles et al., 2009; Lakens and Stel, 2011); exposure to synchronous stimulation enhances the degree of self-other merging (Paladino et al., 2010); and active engagement in synchronized physical and verbal activities boosts actual liking and cooperation (Hove and Risen, 2009; Wiltermuth and Heath, 2009; Reddish et al., 2013; Launay et al., 2014), as well as pro-social behavior toward an interaction partner (Valdesolo and DeSteno, 2011).

To date, an impressive breadth and variety of studies investigating behavioral coordination has been published. However, there are several fundamental questions about the phenomena, which are currently unanswered. For instance, do the effects of coordination scale up from pairs of people to small and then large groups? With a few exceptions (e.g., Wiltermuth and Heath, 2009; Reddish et al., 2013; Codrons et al., 2014; Tarr et al., 2015), behavioral coordination has mostly been studied in pairs, which makes it difficult to generalize from two people to large groups of people. These studies have also mostly studied the effects of coordinated movement. So one might wonder, does it matter which aspect of behavior is coordinated – speech, posture or gesture – in order to produce particular psychological effects? Finally, are the benefits of coordination restricted to social judgments – attitudes and opinions about other people – or does it also affect cognition and joint action, such as the ability of people to perform a dynamic task together?

First, we will briefly review the current answers we have to these questions with a focus on synchrony as a particular form of behavioral coordination. Then, we present an experiment combining verbal synchrony and group action that attempts to answer some of the unresolved issues. Finally, we will discuss how the results of our study fit in with existing research and which future research directions could be taken to clarify the subject further.

### Synchrony in Groups

Most experimental demonstrations of coordinated behavior focus on pairs of participants, or more commonly, a participant and a confederate who has been instructed to mimic body motions (e.g., Chartrand and Bargh, 1999). Most of the findings reported in the synchrony literature also stem from either experienced or observed dyad interaction. While the findings reported significantly advance our understanding about the circumstances under which synchrony emerges and the effects it has, generalizations from pairs to groups can be problematic. It is therefore crucial to also study synchrony experienced in a group context, as coordinated behavior has played an important role throughout history and cultures (Haidt et al., 2008), and has lost none of its significance. Even today, soldiers are still drilled to march in synchrony during their education and parades all over the world, synchrony is frequent in dance and sports, and collective chants take place during rituals, demonstrations, and religious ceremonies to name but a few examples.

One of the reasons for the lack of group studies in relation to synchrony and behavioral coordination more generally is that it is almost certainly difficult to get more than one or two participants into the lab at the same time, or having to coordinate multiple confederates simultaneously. A second possible reason is that group data is often very noisy and challenging to make sense of. In spite of these difficulties, a few studies have been published, which have looked at the effects of synchrony experienced in bigger groups. These studies report that synchrony increases aggressive behavior toward an outgroup and obedience to a leader (Wiltermuth, 2012a,b), while at the same time it increases ingroup affiliation (Tarr et al., 2015), and cooperation (Reddish et al., 2013). Similarly, in a recent study we found that the amount of distributed coordination naturally emerging over time in a choreographic task, which facilitated synchrony without instructing it, predicted how much group members liked each other and the group as a whole, and how much they conformed to each others opinions (von Zimmermann et al., under review). While these studies suggest that synchrony at the group level has similar effects as it has at the pair level, the evidence is still sparse.

### Synchrony across Different Behaviors

Does it matter which kind of behavior is synchronized, or simply that the same action happens at the same time between two or more people? The literature is not clear on this point, as the many skeins of behavioral coordination that have been discovered are isolated in different disciplines, different tasks and types of interaction, different measures and means of analysis. Social psychologists may study mimicry between gestures, ecological psychologists the rhythmic entrainment of body sway, and psycholinguists the repetition of grammatical forms. These differences are important to the scientists, but are they important to the psychological outcome of behavioral coordination?

We wanted to investigate whether the effects of movement coordination reported in the literature would also result from verbal coordination alone. Speakers have been found to possess a remarkable ability to speak in synchrony with one another, without any practice or detailed instructions (Cummins, 2011). Perhaps unsurprisingly then, chanting, or joint speech, can be observed in every human culture, and as a means of storing and passing on information, it predates the written word (Cummins, 2013). It has been speculated that when a group moves and chants together, this will help to increase group affiliation and improve the group’s coordination (McNeill, 1995). However, it is not clear if verbal behavior alone will produce positive effects that will spread over to forms of movement coordination.

### Effects of Synchrony on Cognition and Action

While some studies have investigated how social and cognitive influences, such as the socially undesirable actions of others (Miles et al., 2010a), a cooperative versus a competitive context (Schmidt and Richardson, 2008), or a pro-social mindset in comparison to a pro-self focus (Lumsden et al., 2012), affect the emergence and stability of synchrony, the majority of empirical measures of behavioral coordination are concerned with the positive feelings that an individual will have toward the person or group with whom they are coordinating (von Zimmermann and Richardson, 2015). Sometimes these effects are measured by ratings and judgments the individual makes about the joint performance or likeability of an interaction partner or group, or the degree of similarity and closeness they feel toward them. At other times the effects are measured by decisions the individual makes about sharing resources or opting to cooperate with the group even if that means to personally sacrifice.

In addition to social outcomes, it is possible that behavioral coordination leads directly to changes in cognition and action. Discussions about the evolution of behavioral coordination often focus less on the advantages of liking and positive feelings in a group, and more on the adaptive value of being able to act as a coherent group, planning and executing a hunt, for example. Performance benefits from behavioral coordination are rarely studied, however, with one exception that we are aware of. Valdesolo et al. (2010) found that synchronous rocking in a chair increased the perceptual sensitivity of participants, which helped them perform better on a subsequent joint action task, in which they had to coordinate their movements with those of an interaction partner. Their findings suggest that there is indeed a synchrony-action as well as a synchrony-cognition link and that sharing the specific skill of synchronization might influence the execution of other joint tasks by enhancing cooperative and collaborative skills. Yet, the empirical evidence for the idea that synchronizing behavior at one time improves future action coordination is still sparse and calls for more extensive scientific investigations.

Furthermore, even though there is some evidence that hand movements performed in synchrony enhanced participant’s memories for an interaction partner’s utterances and facial appearance (Macrae et al., 2008), the benefits of synchronized activity on memory are not well-established, yet. More specifically, the possible benefits of collective speech on memory seem to have been overlooked entirely (von Zimmermann and Richardson, 2015). This is interesting since collective speech is employed in educational settings in which remembering the spoken word is important such as in schools or churches. On top of that, one could speculate that national anthems, songs sung at sport events, or slogans shouted during demonstrations are remembered not only because people are exposed to them frequently, or because they are memorable, but also because they are almost exclusively associated and performed with the collective.

### Verbal Coordination, Groups, and Action

In our experiment, groups of 20–30 participants either read a list of words out loud together or individually. Participants reading single words in unison is quite different to the coordinated, spontaneous joint speech that one finds during demonstrations or at a football game. However, it is a first approximation, and allowed a close comparison with participants in the asynchronous speech condition. Those people read the same words out loud, but started at different places in the list, and so spoke out of time with each other.

**FIGURE 1:**
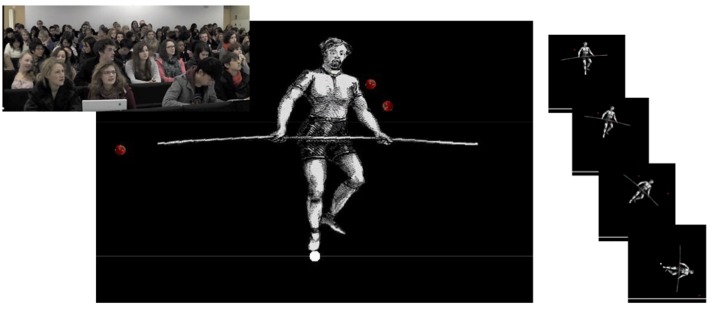
**The tightrope game (taken from Richardson et al., 2011)**.

After reading for around 2 min, participants played a group video game in which they used audience response handsets to jointly control a tightrope walker and keep him upright (Richardson et al., 2011). Following the game (**Figure 1**), participants were asked to recall as many words as possible from the list, and rate their feelings toward their group. Our hypotheses were that those in the synchronized reading condition would perform better as a group in the action task, they would remember more words from the list, and have increased feelings of group affiliation.

## Materials and Methods

### Participants

In exchange for course credit, 215 participants from UCL participated in this study (*M* age = 18.85, *SD* age = 0.90, Number of Males = 35). They were run in eight groups of between 23 and 34 people as part of a lab demonstration course. The participants were informed that this was research on the ‘effects of memory retrieval’ and were unaware of the true research hypothesis until after the experiment was complete.

### Ethics Statement

Ethical approval was obtained from the UCL Research Ethics Committee. All participants consented to taking part in this experiment and were fully debriefed upon completion of the study.

### Apparatus and Stimuli

Each participant was given a *Turning Technologies* audience response handset. Button presses were sent to a USB receiver plugged into a MacBook. These responses were sent to the tightrope game, developed by *Delosis.* The MacBook was connected to a projector, which displayed the game on a large screen that everyone could see.

In the game, participants saw a man holding a pole, balancing on a rope (**Figure 1**). Each time one of the participants pressed either 1 or 3 on their handset, it sent a very small nudge to the tightrope walker, sending him to the left or right. The size of individual nudges depended on the number of people playing, such that the strength of all nudges added together would be the same across games with different numbers of people. The game was made harder by tomatoes that were fired from the sides of the screen, destabilizing the tightrope walker. They appeared at random and their frequency varied to change the difficulty of the game. The movements of the tightrope walker and the appearance of the tomatoes were governed by a physics engine that accounted for the size and position and momentum of the objects.

**FIGURE 2:**
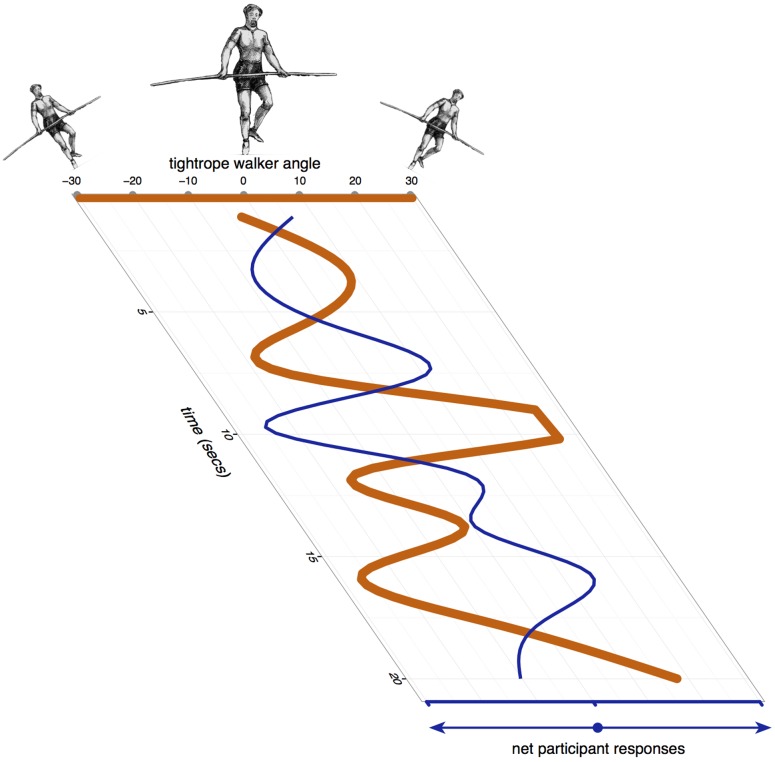
**Example of a game played from a single trial of an experiment**. The thick orange line shows the angle of the tightrope walker, and the thin blue line shows the net left or right nudge from a group of participants as they try to keep him upright.

A game ended when the tightrope walker fell off the rope, or participants successfully kept him upright for 30 s. **Figure 2** shows the tightrope walker’s angle and the net response from the audience across 20 s of one of the games in our experiment.

### Procedure

Participants were randomly allocated to groups, and each group was assigned to the synchronous or asynchronous speech condition. Participants were given a list of 54 words, split into three columns. They were told to read them out loud, completing two cycles of the entire list. In the synchronous condition, participants were instructed to start at the top of the page with the first word and read the words at the same time as each other. In the asynchronous condition, participants were first given a number between 1 and 3. They were told to start reading at the top of the first, second, or third column, respectively. Since participants were numbered consecutively where they sat, participants sat next to each other always started in different places.

Once participants had read through the list twice (which typically took around 100 s) they were introduced to the tightrope game. They were allowed a practice session with no tomatoes being fired as we explained how they could control the tightrope walker. Then they played five games with monotonically increasing rates of tomatoes being fired at them. If the tightrope walker fell off before 30 s, the game was restarted, until participants were able to complete a total of 30 s.

After playing the game, participants filled in a worksheet. In 60 s they wrote down as many of the words as they could remember from the list that they had read out previously. Then they responded on a 7-point Likert scale from ‘strongly disagree’ to ‘strongly agree’ to the following statements, designed to assess participants’ positive feelings toward their group:

(a) During the video game I felt that my group performed well.
(b) I enjoyed playing the video game together with my group.
(c) During the video game I experienced a feeling of togetherness with my fellow group members.
(d) I felt that my group acted like a team while we were playing the video game.

## Results

### Memory and Affiliation

**FIGURE 3:**
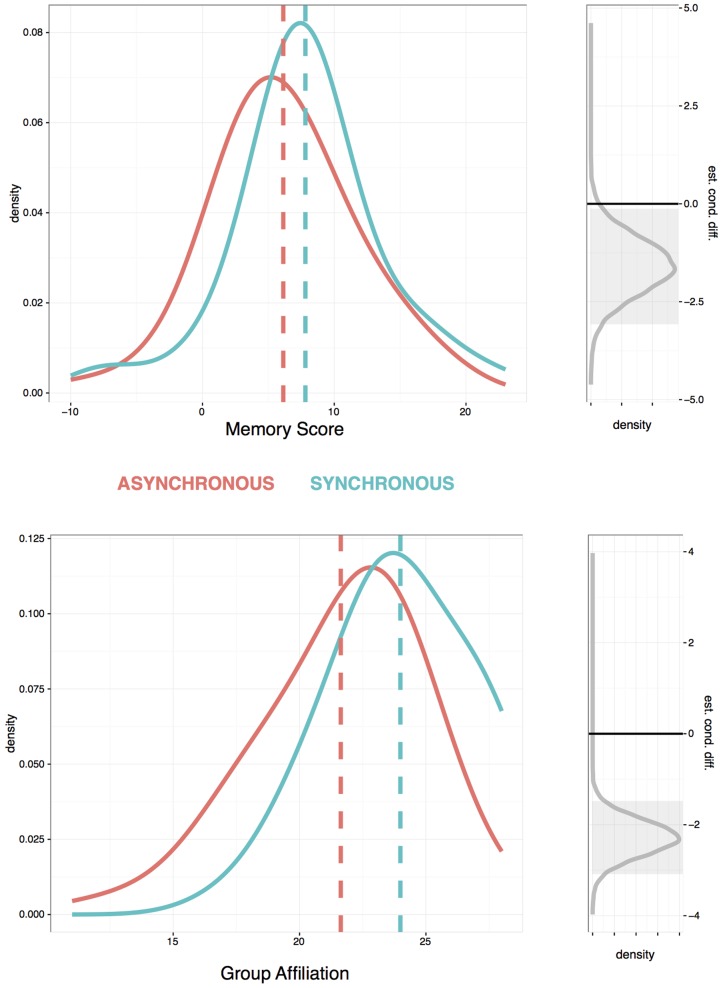
**Participants’ scores on memory for words (top) and group affiliation ratings (bottom).** Red and blue lines show the distribution of scores in the asynchronous and synchronous chanting conditions. Gray lines show the Bayesian estimate of distribution of the difference between conditions and gray areas show their 95% credibility intervals.

Participants in the synchronous conditions scored better on the memory test and felt more affiliation toward their groups, as shown in the two distributions plotted in **Figure 3**. For a memory score, we counted the number of words that participants correctly recalled minus the number that they incorrectly recalled. For every participant, the averages of the four affiliation items were calculated. Affiliation ratings for the synchronous groups (*M* = 25.22, *SE* = 0.39) were higher than for the asynchronous groups (*M* = 22.20, *SE* = 0.51), and memory scores for the synchronous groups (*M* = 6.96, *SE* = 0.93) were also higher than for the asynchronous groups (*M* = 4.15, *SE* = 0.70). Conventional t-tests found significant differences between conditions for the memory scores [*t*(212) = -2.20, *p* = 0.029] and affiliation ratings [*t*(212) = -5.88, *p* < 0001]. The BayesFactor package (Morey and Rouder, 2015) in R was used to estimate the odds of differences between the conditions, plotted on the right of **Figure 3**. For both, memory score and rated group affiliation, an estimated difference of zero between the conditions lay outside the 95% credibility interval (Kruschke, 2010), giving strong evidence in favor of an effect of condition. Participants in the synchronous speech condition remembered more words than participants in the asynchronous speech condition and they also expressed higher levels of liking for their group.

### Tightrope Game Performance

**FIGURE 4:**
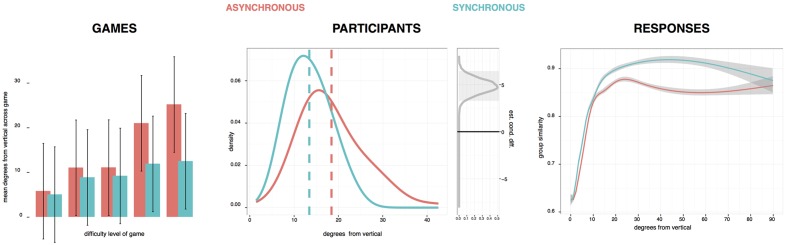
**The distance of the tightrope walker from the vertical at different levels of analysis: averaged across all games; averaged when each participant clicked; and plotted against the similarity between participants’ response.** Groups that chanted asynchronously are in red, those that chanted synchronously are in blue.

We analyzed performance on the tightrope game at three levels, as shown in **Figure 4**. At the broadest level, groups in the two chanting conditions succeeded at the game to roughly equivalent degree, measured by how close to upright they kept the tightrope walker. At the lowest difficultly level, all groups managed the task without having to restart, whereas at the highest level there were 1.3 restarts on average. However, there was not a significant effect on the number of restarts by difficulty level, condition, nor an interaction (all *F*s < 1). Yet, looking in more detail at *how* they played the game, participants in the synchronous chanting condition tended to make a response more readily when the tightrope walker was closer to the vertical, and at each moment in time, their responses tended to be more homogenous within the group.

For each game, we calculated the average distance of the tightrope walker from the vertical in degrees. We ran an ANOVA with difficulty level and chanting conditions as factors, but there was no main effect of condition [*F*(1,6) = 1.1], only a marginally significant effect of difficulty level [*F*(1,59) = 3.51, *p* = 0.08], and no significant interaction [*F*(1,6) = 0.83]. To analyze individual participants’ behavior, we calculated the average distance of the tightrope walker from the vertical at each moment the participant made a response. Participants in the synchronous condition made responses when he was approximately 5° closer to vertical. Bayesian analysis showed that the 95% credibility interval for this difference was above zero, which was also reflected by a significant *t*-test on the condition means [*t*(192) = 6.43, *p* < 0.0001].

Finally, we analyzed individual responses, calculating the proportion of identical responses that occurred 250 ms before and after each one. For each chanting condition, we plotted this measure of group similarity against the distance of the tightrope walker from the vertical. As can be seen in the final plot of **Figure 4**, when he was close to vertical, group similarity in responses was low, as participants were nudging him to both the left and right to keep him balanced. As he veered away from the upright, groups responses increasingly became more similar, as it was more apparent which direction he needed to be nudged in order to right him. However, the two chanting conditions differed in this regard. As shown by the non-overlapping confidence intervals, from around 10° onward, responses in the synchronous group were more similar to each other moment by moment. A Bayesian analysis confirmed that between 10 and 70°, the 95% credibility interval for this difference between conditions was above zero.

In summary, there is evidence that reading out the list of words together had an effect on participants’ behavior in a task of group coordination. When the results were analyzed at the level of games and groups there was only a marginally significant effect of chanting conditions. However, when individuals’ responses were analyzed, we found that those in the synchronous condition more readily made responses as the tightrope walker deviated from the vertical, and once he passed 10° from the vertical, responses amongst the synchronous group were more similar to each others.

## Discussion

With our experiment we wanted to expand on already existing synchrony and behavioral coordination literature in three ways. First, we wanted to see if the affiliative effects generally reported in pair studies scale up to larger groups. While some studies have reported that synchronized movement in small groups increased liking amongst group members (e.g., Reddish et al., 2013; Tarr et al., 2015), we also found that members of large groups reported to feel closer to each other after they had chanted together in synchrony. The finding that behavioral synchrony can lead to interpersonal liking and rapport seems to therefore hold true also for much larger groups than previously reported on. This might not come as a surprise since human beings have engaged in synchronous movement and collective speech as part of rituals for centuries with important social consequences: Participation in collective rituals promotes social cohesion and thereby strengthens individuals’ attachments to each other and the group, making effective group action possible (Whitehouse and Lanman, 2014). Respectively, research has shown that rituals not only significantly increase ingroup affiliation in comparison to non-ritualistic group activities (Wen et al., 2016), but those rituals, which include synchronous behavior, lead to increased liking and cooperation within a group (Fischer et al., 2013).

Second, we wanted to investigate if verbal synchrony alone is sufficient to induce the affiliative effects of behavioral coordination generally observed. Our groups were only instructed in relation to their verbal coordination, but no statements were made with reference to movement. This means that in theory, through the coordination of their verbal articulations, group members might have also spontaneously coordinated their postural movements (Shockley et al., 2009), and possibly even started sharing physiological dynamics such as heart rate (Fusaroli et al., 2016). While we cannot completely exclude this as a potential alternative explanation of, or at least mediating influence on our findings, we do not believe that any kind of physical coordination, which might have occurred, would have been strong enough to explain our results. In contrast to other experiments, which reported spontaneous coordination of movement or physiological functions, our participants were seated in rows next to and behind each other and did not have direct eye contact with one another. Except for chanting together they also did not interact with each other in any other way before moving on to playing the tightrope game. We are thus confident that verbal synchrony – as the prevalent form of coordination in the experiment – was the main mechanism, which lead to significant changes in our participants. Respectively, individuals’ ratings of their perceived affiliation with the group and their groups performance increased in the synchronous condition. Joint speech, like joint movement, allows interaction partners to construe a shared representation of the world, in which intentions become aligned and common ground is established (Cummins, 2014). Like protestors chanting the same slogan together, demonstrating an extreme form of alignment with respect to the world (Cummins, 2014), the participants in our synchronous speech condition probably experienced higher levels of alignment than those participants, who were reading the words out asynchronously. Through coupling their actions during the joint speech task, participants established a common goal with affiliative, cognitive and coordinative consequences.

Third, we were curious to see if synchronous behavior would also affect action and cognition in addition to the social effects often observed. In other words, we wanted to find empirical evidence for the hypothesis that there is a synchrony-action link, that group members, who have previously synchronized with one another, will be better coordinated in a subsequent task. Our evidence supports this idea. Groups overall seem to do better on a coordination task after their members have engaged in synchronous behavior, at least at the harder levels of task difficulty. Why might this be the case? To successfully coordinate behavior and synchronize, people need to anticipate each other’s behaviors (Sebanz et al., 2006; Konvalinka et al., 2010). In this respect, it has been argued that perceiving another’s movements, for example, activates one’s own action system for that same movement, which increases the likelihood for a matched action to occur (Brass et al., 2001). This suggests a tight neural link between perception and action, which could extend to the development of shared representations of a joint action task and of self and other (Hurley, 2008; Kirschner and Tomasello, 2009). While an increase in self-other overlap is said to foster social bonds (Galinsky et al., 2005), one could speculate that participants in our synchrony condition were able to develop a shared representation of the chanting task and each other, which then influenced not only their feelings for each other, but also improved their coordinative skills in the tightrope game.

In this study, however, not only did we find a synchrony-action link, but also a synchrony-cognition link: Participants who had chanted words collectively, rather than reading them out loud by themselves, remembered more of these words at the end of the experiment. With the present data, of course, we can’t judge whether the reason for the memory improvement in the synchronous condition was because the asynchronous chanting was a distraction to participants, and this caused them to encode fewer words in the first place, or because of motivational benefits from higher perceived affiliation with the synchronous group, or because of a general performance boost that mirrored the improved performance in the balancing task. In spite of this limitation, our results seem to be in line with the findings from two other studies, which looked at the relationship between synchrony and memory, albeit in relation to social information. Synchronous movement was reported to enhance people’s attention for each other during a social exchange, enhancing memory for another’s verbalizations as well as their facial appearance (Macrae et al., 2008). Comparing the memory performance of participants, who listened to words over headphones, while engaging in arm curls together with a confederate either in-phase or in the less stable anti-phase coordination, produced a memory advantage for self-related in comparison to other-related information in the anti-phase coordination, whereas this effect was eliminated when participants had moved in-phase with the confederate (Miles et al., 2010b). The findings from our study suggest that synchronous actions might not only influence memory in relation to social information, but more generally as well. This, however, needs to be tested more rigorously in the future.

A diverse set of researchers have come to the realization that perception, action and cognition cannot be fully understood by investigating single individuals (e.g., Sebanz et al., 2006; Barsalou et al., 2007; Robbins and Aydede, 2009). Studies of situated cognition show that cognition ‘in the wild’ is intimately linked not only to representations of the external world, but also to the cognitive processes of others. For example, Hutchins (1995) observed the ways that navy navigators would distribute cognitive processes between themselves by using external tools and representations, such as maps and notations. In the past few years, experimental methods have also started to reveal the cognitive mechanisms involved in the joint activity of two people engaged in parallel tasks (Sebanz et al., 2006), talking to each other (Richardson D. C.et al., 2007), or just silently looking at pictures, changing their gaze patterns because of the knowledge that someone is looking at the same thing (Richardson et al., 2012). Knoblich and Jordan (2003) gave a detailed analysis of the way that two people coordinate their actions: To be successful, participants had to anticipate both the movements of the objects in the game and the actions of their partner. It is possible that chanting together in our experiment helped participants to anticipate each other’s actions and thereby facilitated coordination in the tightrope walker game. However, it becomes clear that no explanation at this point goes beyond speculation. It will therefore be an interesting task in the future to study how perception, cognition and action are linked in social situations, which involve more than two people, and what the exact mechanisms are, which could explain a synchrony-action link.

Behavioral coordination is often portrayed as something that binds people together, evoking positive and pro-social feelings toward interaction partners. However, there is more to coordinated joint action than hugs. For example, while synchrony, like mimicry (Chartrand and Bargh, 1999) often increases rapport and cooperation, sometimes it has quite different results. In two studies, Wiltermuth (2012a,b) showed that synchrony can lead to aggressive behavior and destructive obedience. People who had just bonded with one another through synchronous action were more likely to comply with each other’s requests, even if this entailed to engage in aggressive behavior toward others, such as administering a noise blast to another group of participants, or killing sow bugs at a leader’s request (Wiltermuth, 2012a,b). These studies support the idea that physical synchrony does not exclusively lead to pro-social, but also to anti-social and destructive behavior. There seems to be a dark side to the phenomenon, and verbal synchrony seems to have comparable effects. Spectators at a football game who had engaged in collective chanting during the game reported higher levels of aggression than those who had not chanted (Bensimon and Bodner, 2011).

## Conclusion

Anthropologists and historians have long argued that acting together in time influences group cohesion and group action. In our experiment, large groups of people, who had engaged in collective speech, acted better together in a subsequent task, displayed improved cognitive functions, and liked each other more. Although we were able to explore the scope of behavioral coordination in our experiment, there is one significant question about the directionality of the effects we found, which we cannot answer with our findings. Does synchrony increase group affiliation and thereby improve cognition and action, or does synchrony increase group performance and this improvement increases the attraction to the group? No matter what the answer to this question is, the New Zealand rugby team should keep performing the haka prior to important games, as it might be an important part of their success strategy.

## Author Contributions

JvZ developed the initial experimental design. Both authors performed the testing and data collection together. DR performed the data analysis. Both authors contributed equally to the writing of this manuscript and approved the final version for submission.

## Conflict of Interest Statement

The authors declare that the research was conducted in the absence of any commercial or financial relationships that could be construed as a potential conflict of interest.
